# Pulse Dosing of Antibiotic Enhances Killing of a *Staphylococcus aureus* Biofilm

**DOI:** 10.3389/fmicb.2020.596227

**Published:** 2020-11-09

**Authors:** Kirsten J. Meyer, Hannah B. Taylor, Jazlyn Seidel, Michael F. Gates, Kim Lewis

**Affiliations:** Department of Biology, Antimicrobial Discovery Center, Northeastern University, Boston, MA, United States

**Keywords:** biofilm treatment, *Staphylococcus aureus*, antibiotic tolerance, persister resuscitation, intermittent dosing, periodic dosing, pulse dosing, oxacillin

## Abstract

Biofilms are highly tolerant to antibiotics and underlie the recalcitrance of many chronic infections. We demonstrate that mature *Staphylococcus aureus* biofilms can be substantially sensitized to the treatment by pulse dosing of an antibiotic – in this case, oxacillin. Pulse (periodic) dosing was compared to continuous application of antibiotic and was studied in a novel *in vitro* flow system which allowed for robust biofilm growth and tractable pharmacokinetics of dosing regimens. Our results highlight that a subpopulation of the biofilm survives antibiotic without becoming resistant, a population we refer to as persister bacteria. When oxacillin was continuously present the persister level did not decline, but, importantly, providing correctly timed periodic breaks decreased the surviving population. We found that the length of the periodic break impacted efficacy, and there was an optimal length that sensitized the biofilm to repeat treatment without allowing resistance expansion. Periodic dosing provides a potential simple solution to a complicated problem.

## Introduction

Biofilms, multicellular communities of bacteria, can be highly tolerant to antibiotics (Spoering and Lewis, 2001). Inside the biofilm, bacterial cells are closely packed together and surrounded by an extracellular matrix. The tolerance of biofilms to antibiotics is linked to many physiological factors. Low nutrient and oxygen availability, upregulation of innate resistance factors, cell-cell signaling, heterogeneity in population gene expression profiles, the extracellular matrix, stress responses, and cell dormancy have all been implicated (Mah et al., 2003; Walters et al., 2003; Rani et al., 2007; Mulcahy et al., 2008; Macia et al., 2011; Nguyen et al., 2011; Kirby et al., 2012; Koch et al., 2014; Van Acker et al., 2014; Stewart, 2015; Ciofu et al., 2017; Hall and Mah, 2017; Stewart et al., 2019). Moreover, the extracellular matrix protects bacteria in biofilms from clearance by the immune system (Otto, 2018; Yan and Bassler, 2019). Biofilm-based infections are a major health burden, leading to treatment failure and chronic relapsing infections (Lewis, 2008, 2019). This is exemplified by the endless rounds of treatment and substantial morbidity and mortality associated with *Pseudomonas aeruginosa* biofilms in cystic fibrosis (Costerton et al., 1999) and staphylococcal biofilms on prosthetic joint and device-related infections (Otto, 2018).

Biofilms, even of the same bacterial species, can differ widely in structure and matrix composition depending on environmental conditions. *In vitro* under lab conditions, broth nutrient components and biofilm age strongly impact antibiotic tolerance, with increasing tolerance as the biofilm matures (Anwar et al., 1992; Haagensen et al., 2015). Experiments with *P. aeruginosa* and *S. aureus* have shown that in mature biofilms, oxygen concentration rapidly decreases from the surface of the biofilm to the deeper layers of cells (Xu et al., 1998; Walters et al., 2003; Rani et al., 2007). This correlates with the metabolic activity of cells, with high transcription rates restricted to exposed surface layers, and with the remainder of the biofilm being slow growing or even dormant. These regions of metabolic activity in the biofilm also correspond to the regions of efficacy of antibiotics (Walters et al., 2003; Haagensen et al., 2015; Stewart, 2015). Slow growth and dormancy lead to tolerance with multiple classes of antibiotics as most require active cell processes to corrupt for efficacy (Lewis, 2008; Conlon et al., 2016; Shan et al., 2017). Growth in biofilms enriches for such antibiotic tolerant cells, termed persisters (Roberts and Stewart, 2005; Lewis, 2010; Conlon et al., 2015; Waters et al., 2016). This tolerance due to low metabolic state is distinct from resistance, as at the population level there is no shift in the minimal inhibitory concentration (MIC) of antibiotics. Instead, these cells have the ability to survive a prolonged presence of antibiotic and resume growth following treatment (Balaban et al., 2019).

We and others have reasoned that as persister cells resuscitate post-treatment to generate antibiotic susceptible cells, intermittent or pulse dosing is a viable strategy for reducing the persister burden (Bigger, 1944; Cogan, 2006; Lewis, 2008; Sharma et al., 2015; Carvalho et al., 2018). Within a biofilm, there will be two populations of cells – susceptible and tolerant to the antibiotic. Applying antibiotic will kill the susceptible cells, while the tolerant population remains. Removing the antibiotic in the presence of nutrients will allow a portion of the dormant tolerant cells to resuscitate and transition to be susceptible. Therefore, if antibiotic is reapplied before new persister formation, the new susceptible portion can be removed, and the process can be repeated until all the tolerant population have been resuscitated and killed. Periodic dosing against planktonic batch cultures have found varying effects on persister levels (Bigger, 1944; Sharma et al., 2015; Cogan et al., 2016; Feng et al., 2016), and periodic application of biocides against environmental biofilms has demonstrated the importance of timing for efficacy (Grant and Bott, 2005). Several groups have modeled *in silico* the effects of varying the period of antibiotic dosing against biofilms (Cogan et al., 2013; Imran and Smith, 2014; Zhao et al., 2016; Carvalho et al., 2018), but to our knowledge, *in vitro* experimental studies systematically testing this approach have been lacking. Using *S. aureus* biofilms, we examined this experimentally with oxacillin and found that indeed providing a periodic break from antibiotic was able to dramatically improve efficacy of treatment.

## Materials and Methods

### Reagents and Cell Lines

*Staphylococcus aureus* HG003 containing a chromosomal *lux* operon (*luxABCDE* modified from *Photorhabdus luminescens*) and a chloramphenicol resistance marker (moved by phage transduction from Newman-*lux* strain; Plaut et al., 2013) was used for all experiments. The lux strain had the same growth rate as parent (Supplementary Figure S1A), and was used with the intention of luminescence being a read out for biofilm metabolic activity (Supplementary Figure S1B), but the noise from the change in environmental conditions (e.g., temperature and oxygen) on luminescence quantification in the biofilm was unfortunately greater than the experimental signal. Single colonies were streaked on to brain heart infusion (BHI; BD BBL) agar plates from glycerol stocks, and were put into overnight culture in BHI broth (shaking 200 rpm, 37°C), before being diluted and used for experiments. Antibiotic stock solutions were stored at −20°C: oxacillin (TCI), vancomycin (Sigma), and gentamicin (Acros Organics) in water; rifampicin (Sigma) in DMSO. Oxacillin MIC was 0.125 μg/ml.

### Biofilm Flow System

Input bottles, silicone tubing (Tygon 3/32" ID × 5/32" OD, Masterflex 3/16" ID × 5/16" OD, and Ismatec Tygon S3 0.89 mm ID, Cole Parmer), glass segments (7 cm long, Borosilicate 4 mm ID × 6 mm OD, Amazon), and waste bottles were autoclaved for sterilization. Tubing segments were connected *via* polypropylene Luer connectors or barbed fittings (Cole Parmer). Sterile 14G polyurethane I.V. catheters (SurFlash Terumo) were cut into 1 cm segments (approximate outer and inner surface area combined 120 mm^2^), transferred to a microcentrifuge tube, and submerged in FBS (ATCC) overnight at 37°C, to promote bacterial adherence. Individual segments were then transferred to 1 ml of OD_600_ 0.01 *S. aureus* suspension in BHI + 1% glucose broth in microcentrifuge tubes, and were incubated for 24 h at 37°C. The medium was supplemented with glucose to stimulate biofilm formation and to rapidly achieve mature and tolerant biofilms (Mack et al., 1992). Catheters were transferred to fresh BHI + 1% glucose for 24 h, and then placed in triplicate inside sterile glass segments. The glass segments were connected inline to the flow system *via* tight sealing silicone tubing (ID 3/16") and Luer connectors. The tubing portion containing the glass segments (hosting the catheters) were set inside a 37°C incubator. The tubing lines and glass segments were filled with BHI + 1% glucose from input bottles *via* peristaltic pumps, and the flow rate was set to 0.1 ml/min. This regimen was maintained for 16–21 h, and then, antibiotic treatment was initiated by the addition of antibiotic to input bottles. To achieve periodic regimens, input bottles were either changed to antibiotic free BHI + 1% glucose for set intervals of time, or antibiotic was steadily diluted out of input bottles by the addition of fresh broth at the same rate as liquid removal (0.1 ml/min). Syringe pumps (New Era) controlled by programmable electronic timers (Cole Parmer) were used to regularly inject antibiotic into input bottles to achieve dosing schedules. At the end of an experiment, glass segments were disconnected from tubing, catheters removed, and rinsed in saline (0.9% NaCl). Catheters were then sonicated in 1 ml of saline for 5 min, followed by 30 s of vigorous vortexing, and this was repeated three times to disrupt biofilms. The resulting suspension was serially diluted by 10x dilutions and plated on BHI agar plates (in duplicate), then incubated at 37°C, to enumerate colony forming units (CFUs). CFU plating was performed on BHI alone or BHI containing antibiotic to establish resistance expansion. Plates were examined for CFU 24 and 72 h after plating.

### Scanning Electron Microscopy

Catheters were removed from the flow system and were rinsed in 1 ml saline. Catheters were left intact or split using a razor, and were moved to a solution of 2.5% glutaraldehyde in 0.1 M sodium cacodylate containing 0.15% alcian blue and 0.15% safranin O for 24 h at 4°C for fixing and staining. Catheters were washed in 0.1 M sodium cacodylate for 10 min, infiltrated with 1% osmium tetroxide for 30 min, and then washed three times in 0.1 M sodium cacodylate. Biofilms were dehydrated by sequential submersion in 30, 50, 70, 85, and 95%, and then 100% ethanol for 5–10 min at each level. Submersion in absolute ethanol was repeated three times, and then the catheters were placed in a SAMDRI-PVT-3D (Tousimis) for critical point drying using liquid CO_2_. Catheters were mounted on aluminum sample mounts using double-sided conductive carbon adhesive tape and sputter coated with 5 nm of platinum (Cressington 208HR). They were imaged on a Hitachi S-4800 SEM at 3.0 kV.

### Phamacokinetic Confirmation

Samples were taken at intervals from the input bottles, or from the tubing line just prior to glass segments, to check oxacillin concentration. Oxacillin concentration was measured using a bioassay – samples were serially diluted in 96-well plates in BHI and incubated with *S. aureus* OD_600_ 0.001 at 37°C overnight (static), and compared to a standard curve run in parallel with known oxacillin levels. Turbidity was examined by eye and OD_600_ measurement (Biotek Synergy H1), and oxacillin concentration for samples was calculated by multiplying 0.5xMIC concentration (from the standard curve) by the dilution factor of samples that allowed growth to occur. The assay was run on 7 separate days with a total of 15 standard curves. Across days the standard curves displayed a mean MIC of 0.14 μg/ml, SD 0.04 μg/ml, *n* = 7, and a within day mean SD of 0.01 μg/ml (two standard curves differed in their MIC by 2-fold on 1 day, all other standard curves run on the same day shared the same MIC).

## Results

### Catheter Biofilm Flow System

**Figure 1:**
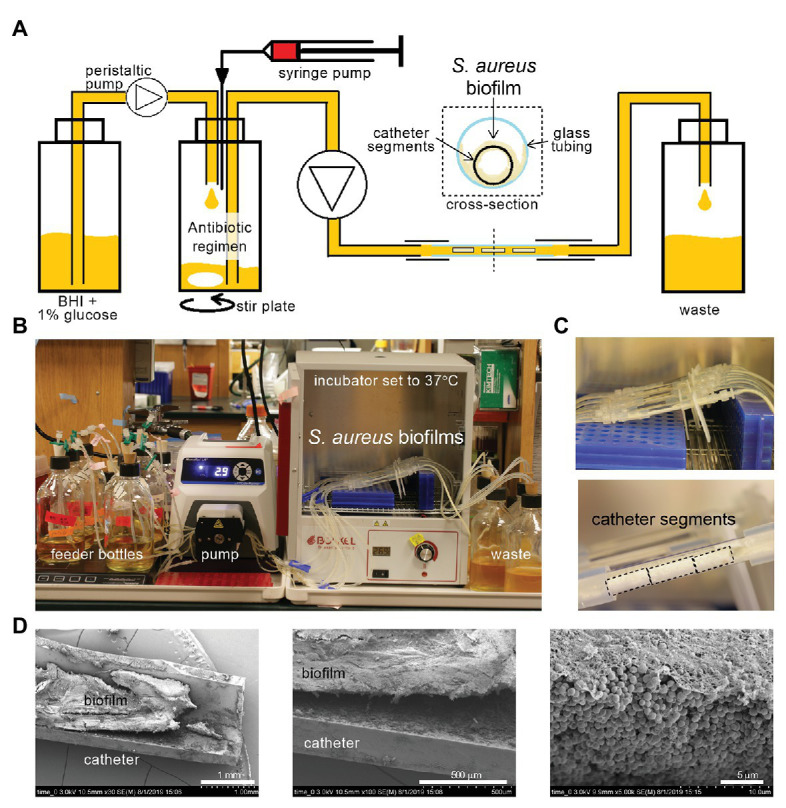
A tractable constant flow system for *Staphylococcus aureus* biofilms. **(A)** Schematic of the flow system. Antibiotic regimens are created in a central bottle *via* injection syringe pumps and dilution with fresh medium [brain heart infusion (BHI) supplemented with 1% glucose], and pumped continuously through a piece of glass tubing containing three 1 cm catheter segments hosting *S. aureus* biofilms, then to a waste container. **(B)** Photograph of one experiment set-up with bottles, tubing, peristaltic pump, glass tubing, and waste bottles. The glass tubing sections hosting the biofilms on catheters are placed inside a 37°C incubator. **(C)** Close up of the glass tubes from **(B)** show thick white *S. aureus* biofilms. Black dotted lines outline catheter segments. **(D)** Catheters were removed from the flow system (Day 0, after 16–21 h of BHI + 1% glucose flow), halved longitudinally, and imaged by scanning electron microscopy. A dense mat of *S. aureus* biofilm can be seen inside the catheter.

To robustly compare the effect of different antibiotic dosing regimens on biofilms, we desired a system with constant liquid flow, so that nutrients could be continually provided and antibiotics adjusted without physical disruption to the biofilm. We began by using the CDC bioreactor (Goeres et al., 2005), but the large volume of medium required for experiments led us to design a smaller system for higher throughput. Catheter segments, 1 cm long, were chosen as the vehicle for the biofilms, providing a reproducible surface area and medically relevant substrate (Kadurugamuwa et al., 2003). The segments were coated with FBS, then inoculated with methicillin-sensitive *S. aureus* (HG003-*lux*, Supplementary Figure S1) in microcentrifuge tubes, and biofilms were allowed to mature for 48 h in rich medium (BHI + 1% glucose). The biofilm-carrying catheters were then placed in triplicate inside glass tubing, and were subjected to a constant flow of medium (Figures 1A,B). All biofilms first received nutrients for 16–21 h (BHI + 1% glucose, 37°C) to allow adjustment to flow conditions and for final maturation, then antibiotic regimens were applied (Day 0). The catheter segments developed robust biofilms, which could be seen by eye (Figure 1C; Supplementary Figure S1B). Scanning electron microscopy revealed dense packing of *S. aureus* into thick mats, covered with a fibrous matrix, on both the inside and outside of the catheters (Figure 1D; Supplementary Figure S2).

**Figure 2:**
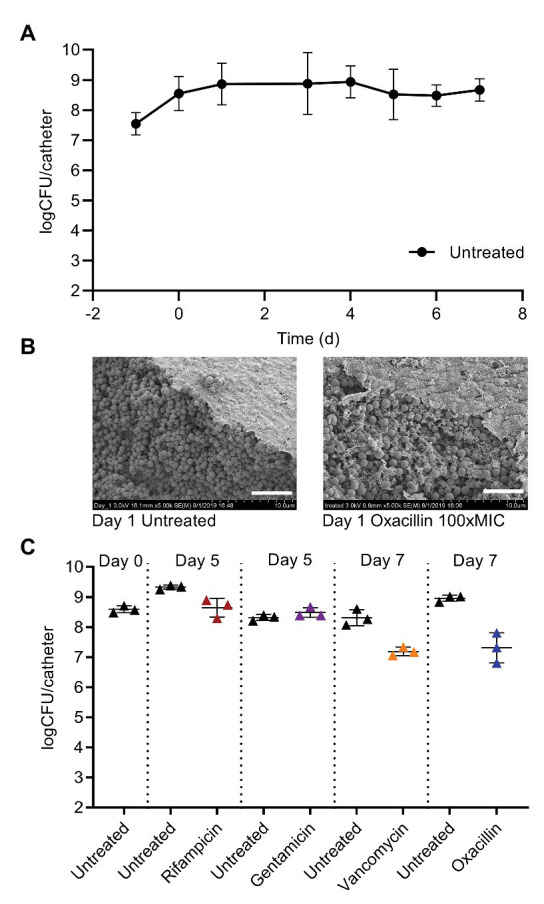
Mature *S. aureus* biofilms on catheters are highly tolerant to antibiotics. **(A)** Catheters carrying 5 × 10^7^ colony forming unit (CFU) of *S. aureus* are placed into the flow system on Day-1, and then fed continuously with BHI + 1% glucose. The CFU per catheter rises to 5 × 10^8^ and stays steady over 1 week of medium flow. Symbols are means of multiple experiments on different days, 2 ≤ *n* ≤ 17, error bars are SD. **(B)** SEM on Day 1 of untreated biofilms, or biofilms treated for 24 h with 12.5 μg/ml oxacillin [100x minimal inhibitory concentration (MIC), planktonic]. White bar, 5 μm. **(C)** Treatment beginning on Day 0 with 2 μg/ml of rifampicin, 50 μg/ml of gentamicin, 100 μg/ml of vancomycin, or 12.5 μg/ml oxacillin (100x the planktonic MICs) and continued for multiple days is ineffective at clearing *S. aureus* biofilms. Data are obtained from representative experiments, and are separated by dotted vertical lines. Symbols represent one catheter, and lines are mean and SD.

To enumerate bacterial density of biofilms, catheters were removed from the glass tubing, rinsed, sonicated, and vortexed in saline, and then the saline suspension was plated for CFU. At Day 0, the biofilms averaged 3.5 × 10^8^ CFU/catheter, and without antibiotic treatment maintained a similar burden over 7 days of providing continuous medium (Figure 2A). The *S. aureus* biofilms grown in this system were highly tolerant to multiple classes of antibiotics. Treatment began on Day 0 with addition of antibiotics to the medium and was provided in a continuous flow past the biofilms. Scanning electron microscopy of biofilms treated for 24 h with oxacillin at 12.5 μg ml^−1^, 100x the planktonic MIC, showed no obvious changes compared to untreated controls (Figure 2B). Even with 7 days of continuous treatment with 12.5 μg ml^−1^ (100xMIC) of oxacillin, there was a maximum of 1.5 log of CFU reduction (Figure 2C). Likewise, the biofilm was stable for multiple days under continuous flow of 100x the planktonic MIC of gentamicin (50 μg ml^−1^), and vancomycin (100 μg ml^−1^; Figure 2C). The biofilm also withstood 100xMIC of rifampicin (2 μg ml^−1^; Figure 2C) but, in this case, the treatment led to the selection for resistance and by Day 5, the biofilm was composed entirely of rifampicin resistant cells. However, for gentamicin, vancomycin, and oxacillin, the treatment did not lead to a substantial increase in resistance compared to untreated biofilms (Supplementary Figure S3).

### Providing Breaks From Oxacillin Can Sensitize Biofilms to Treatment

**Figure 3:**
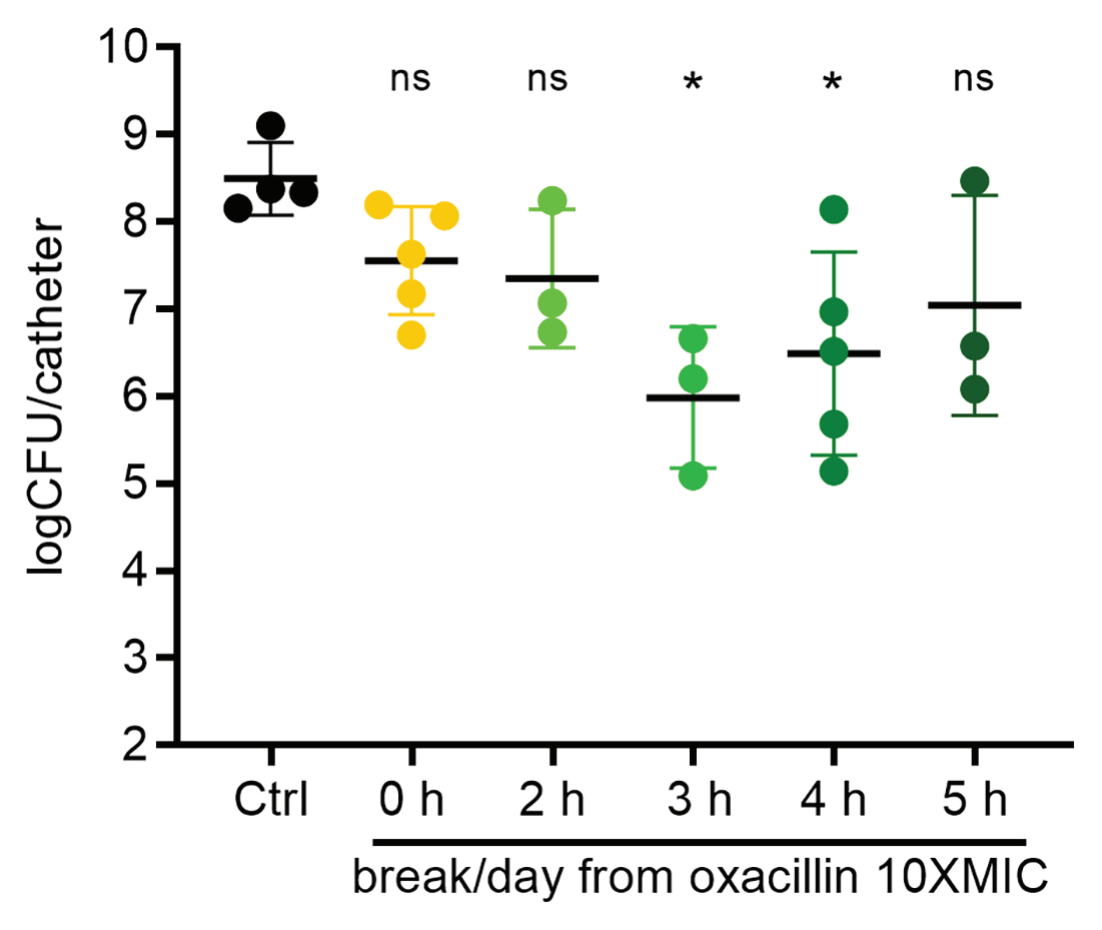
Treatment breaks from constant oxacillin can sensitize biofilms. Biofilms were treated with constant oxacillin at 1.25 μg/ml (10x MIC) for 6 days, with a 0–5 h treatment break each day and plated for CFU. Symbols represent mean of three catheters from one experiment. Lines are means and SD of symbols (repeat experiments). Group means (of the log transformed data) were compared with an ordinary one-way ANOVA, followed by multiple-comparison of all means using Tukey’s test (Prism 8.4.2). The ANOVA found a significant difference between means, *p* = 0.019, and oxacillin treated biofilms with a 3 or 4 h break each day were assessed as different than the control mean with adjusted *p* values of 0.018 and 0.035, respectively. All other comparisons did not reach significance.

For methicillin sensitive *S. aureus*, oxacillin is frequently used in the clinic, and so we chose to focus on oxacillin for studying periodic dosing. We began by providing a regular break from oxacillin each day. *S. aureus* biofilms on catheters were treated for 6 days with either continuous oxacillin (1.25 μg ml^−1^, 10xMIC), or with a 2–5 h break each day by changing the source bottle to antibiotic free broth for the specified interval (Figure 3). Untreated control biofilms had on average 3 × 10^8^ CFU/catheter at the end of 6 days (Figure 3). Continuous oxacillin treatment for 6 days was only able to reduce the biofilms on average 1 log to 5 × 10^7^ CFU/catheter (the 0 h break/day point in Figure 3). A 2 h break each day from the oxacillin made no significant difference to biofilm burdens. Encouragingly, providing a 3 h break each day decreased biofilm survival, with 2–3 logs of the biofilm cleared by the end of the experiment. There was a greater variability in response to breaks of 4 or 5 h; on some experimental rounds, biofilms withstood the treatment, and in others, biofilms were decreased.

### Modeling *in vivo* Half-Lives to Provide a Periodic Break

**Figure 4:**
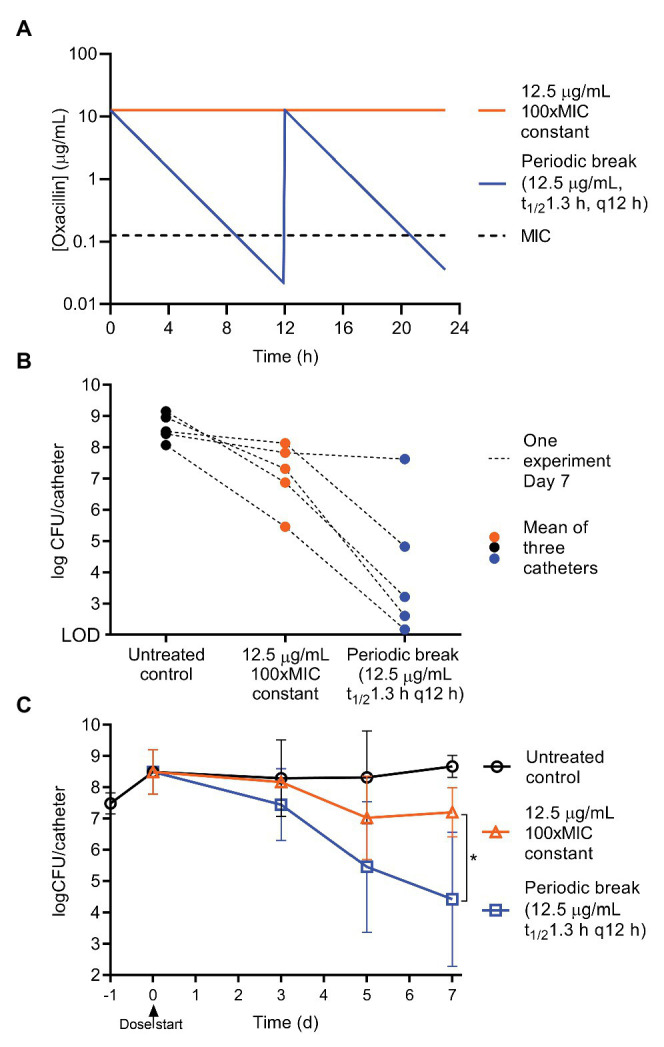
Periodic oxacillin with half-life is more effective than constant regimen. To achieve a 3–4 h periodic break below the MIC, oxacillin was given as a peak of 12.5 μg/ml (100xMIC) with a *t*_1/2_ of 1.3 h (the half-life in mice) every 12 h, and compared to constant 12.5 μg/ml oxacillin. **(A)** Modeled pharmacokinetics. **(B)** Day 7 results from five matched experiments, symbols represent the mean of triplicate catheters, and lines connect the results of treatment groups from one experiment. **(C)** Efficacy of regimens on biofilm CFU over time. Symbols are means of repeat experiments, 3 ≤ *n* ≤ 14, lines SD. Day 7 results (log transformed CFU) for constant and periodic oxacillin were compared with an unpaired *t*-test, *p* = 0.023, with Welch’s correction for unequal variance (Prism 8.4.2).

Typically, antibiotics are given orally or by injection, and the bacteria are exposed to an antibiotic pharmacokinetic (PK) profile that has a peak and then decays over time. To begin to examine the effect of periodic dosing on biofilms using dynamic PK profiles and to enable further translational studies, we simulated oxacillin regimens based on *in vivo* mouse pharmacokinetics. Oxacillin was dosed into the central bottle that feeds the catheters by syringe pump, and then constantly diluted by the addition of fresh medium (Figure 1A) to achieve a half-life of 1.3 h (Jo et al., 2011). To provide a 3–4 h break below the planktonic MIC of oxacillin, 0.125 μg ml^−1^, every dosing period, a regimen was modeled with a peak of 12.5 μg ml^−1^ (100xMIC) given every 12 h, and compared to continuous treatment with 12.5 μg ml^−1^ (100xMIC) oxacillin (Figure 4A, PK sample check Supplementary Figure S4). Encouragingly, in four out of five experiments over 7 days, dramatically improved efficacy was seen in the periodic treatment compared to constant oxacillin, with the periodic regimen clearing 3–5 log more than continuous antibiotic (Figure 4B). In two experiments, the periodic regimen cleared the biofilm almost to the limit of detection (Figure 4B). When this was expanded with further experiments, including endpoints at 3 and 5 days, it appeared that continuous oxacillin (12.5 μg ml^−1^, 100xMIC) cleared 1.5 log of the biofilm between 3 and 5 days of treatment, with an average remaining persister burden of 5% (10^7^ CFU remaining) at 7 days (Figure 4C). In comparison, the periodic regimen caused continuous decline leading to a greater reduction in biofilm burden by Day 7 (Figure 4C). The response was variable, suggesting variation in the slope of biofilm decline between experiments, but the average decline was log-linear and achieved 4 log reduction over 7 days (Figure 4C).

### Break Length Has a U-Shaped Response Curve

**Figure 5:**
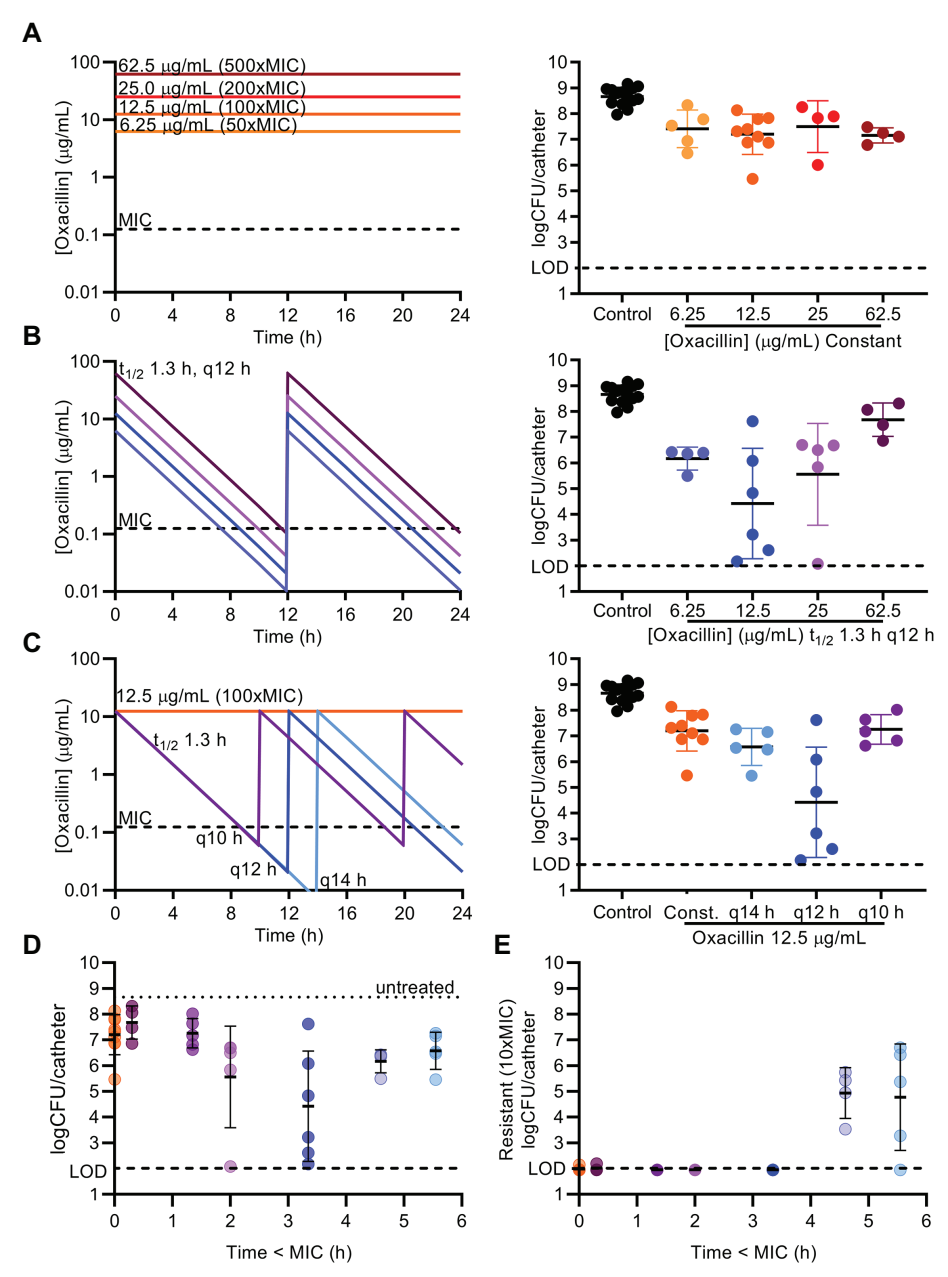
Length of periodic break gives U-shaped dose response curve. Oxacillin was given as constant regimens of 6.25, 12.5, 25, or 62.5 μg/ml (50, 100, 200, or 500x MIC; **A**) or as periodic regimens with a *t*_1/2_ of 1.3 h, and given every (*q*) 10, 12, or 14 h **(B,C)** for 7 days. Programmed oxacillin regimens are depicted on the left. Biofilm suspensions were plated on BHI to determine total CFU/catheter after 7 days (right). Untreated controls, 12.5 μg/ml (100xMIC) constant, or 12.5 μg/ml (100x MIC) q12 h are the same data **(A–C)** visually repeated for purposes of comparison. **(D)** CFU results for each periodic regimen, and 12.5 μg/ml (100xMIC) constant for comparison, are displayed with the time oxacillin was below the planktonic MIC 0.125 μg/ml (Time < MIC) in the programmed conditions. **(E)** Biofilm suspensions were plated on agar containing 10xMIC oxacillin to determine resistant CFU, and only those periodic regimens with the greatest Time < MIC allowed resistance expansion above the limit of detection (LOD). Symbols represent mean of three catheters from one experiment. Lines are mean and SD of symbols (repeat experiments). LOD, limit of detection.

The length of the effective break from oxacillin was modulated by decreasing and increasing the peak concentration of each dose, and by decreasing and increasing the interval of doses, while comparing these regimens to constant oxacillin (Figure 5, PK sample check Supplementary Figure S4). These experiments highlighted the impressive tolerance of mature biofilms – 10^7^ CFU/catheter (5% of untreated biofilms) remained after constant oxacillin at 6.25, 12.5, 25, or 62.5 μg ml^−1^ (50x, 100x, 200x, or 500xMIC, respectively) for 7 days (Figure 5A). Periodic dosing was able to decrease the final biofilm burden, but had a steep U-shaped dose response curve. The optimal periodic regimen was the originally tested peak of 12.5 μg ml^−1^ (100xMIC) of oxacillin given every 12 h, decreasing the biofilm down to 10^4^ CFU/catheter on average (0.006% of the untreated biofilm) after 7 days (Figures 5B,C). Decreasing or increasing the peak concentration by 2-fold was less effective, leaving 10^6^ CFU/catheter (0.2% of the untreated biofilm) remaining (Figure 5B). Shifting the dosing frequency 2 h likewise gave a U-shaped dose response curve (Figure 5C). These results correlate with the time oxacillin was below MIC (0.125 μg ml^−1^) in the modeled regimens (Time < MIC), with the best efficacy achieved with a break from oxacillin of 3–4 h below the MIC (Figure 5D). To assess selection for oxacillin resistance, at the end of every experiment, biofilm suspensions were also plated on plates containing 10xMIC oxacillin. Encouragingly, resistance expansion did not occur in the optimal regimen, and remained below the level of detection (Figure 5E). The regimens providing the longest break, 5–6 h below the planktonic MIC, arising from a peak of 6.25 μg ml^−1^ (50xMIC) oxacillin given every 12 h or 12.5 μg ml^−1^ (100xMIC) given every 14 h, allowed a resistant subpopulation to expand and reach levels of 10^5^ CFU/catheter over 7 days (Figure 5E). Under our conditions of rich medium and very mature biofilms, the optimal break is 3–4 h below the planktonic MIC of the antibiotic to allow reversal of biofilm tolerance but prevent resistance expansion.

## Discussion

Biofilms are a critical problem in health care, leading to chronic recurring infections despite lengthy treatment with antibiotics. Our *in vitro* flow system allowed us to grow mature *S. aureus* biofilms and expose them to a constant flow of nutrients and to various antibiotic dosing regimens (Figure 1). The system is simple and tractable, and minimizes medium use compared to other macroscopic biofilm flow systems available, although experiments remain time-consuming given the impressive antibiotic tolerance of mature biofilms. For motile bacteria, the system could be adjusted with an air break, or equivalent measure, to prevent movement of the bacteria against the direction of flow. A limitation of the system is the use of catheters that likely restrict the flow of liquid through the lumen, and alternative flat substrates could be explored. Despite the complexity of the nature of biofilm tolerance to antibiotics, we found that simply providing a periodic and correctly timed break from an antibiotic (oxacillin) can sensitize a *S. aureus* biofilm to treatment (Figure 4). Although inclination to improve antibiotic efficacy is often to treat with a larger dose of drug for longer, we demonstrate that periodic absence of antibiotic, or the time below a critical threshold (e.g., MIC), can be important for efficacy of regimens against biofilms.

Our study leads to several conclusions. First, it is consistent with previous research and clinical experience and shows that a mature biofilm remains tolerant to antibiotic treatment over multiple days, even while provided with a continuous supply of nutrients (Figure 2C; Parra-Ruiz et al., 2012; Barber et al., 2015). This occurred without selection for resistance in the case of gentamicin, vancomycin, and oxacillin (Figure 2C; Supplementary Figure S3). For simplicity, we refer to this tolerant subpopulation as persisters, despite likely complexity in the mechanisms of tolerance, as these cells withstood antibiotic in the biofilm but when removed from the biofilm were able to regrow in the absence of antibiotic. The levels of persisters in our study were comparable to persister/tolerant subpopulation levels observed in previous studies of mature staphylococcal biofilms and cell-wall inhibiting antibiotics (Anwar et al., 1992; Parra-Ruiz et al., 2012; Barber et al., 2015; Butini et al., 2019). For constant oxacillin, under rich medium conditions, the persister level in our study was remarkably stable across five dose levels from 1.25 to 62.5 μg ml^−1^ (10xMIC–500xMIC) and averaged 5 ± 2% of the total (mean and SD; Figures 3–5).

Second, providing a periodic break from oxacillin for short periods could reverse biofilm tolerance and lead to sensitization to repeated applications of oxacillin (Figures 3–5). Presumably, a subset of the persisters exit dormancy and resuscitate when the antibiotic is not present. Conversely, this suggests that maintaining the presence of antibiotic prolongs dormancy. This may be cellular stress response related, or the direct action of the antibiotic, inhibiting the function of key cellular machinery, which, in the case of oxacillin, is the inhibition of penicillin binding proteins preventing the formation of peptidoglycan.

Third, the extent of sensitization and the rate of removal to a given periodic regimen had considerable variation (Figures 3–5), likely reflecting a heterogeneity in biofilm maturity and response between different experiments even when conditions were replicated. This variation may also have come from the outer and inner surfaces of the catheter experiencing different flow rates.

Finally, under our rich medium conditions, the effective break window from oxacillin was narrow and adjusting the period of oxacillin dosing by 2 h in either directions substantially decreased efficacy. We speculate that too short a break does not allow for adequate persister resuscitation, whereas too long a break allows for population expansion (including resistant cells) and perhaps produces new persisters. This narrow window under our rich medium conditions may also explain the high variability in response to periodic dosing. With the caveat that concentrations in the microenvironment of the biofilm may have been different than in the bulk flow system broth, our models suggest the effective break from oxacillin required 3–4 h of the antibiotic below the planktonic MIC (Figures 3–5). Further research is needed to explore optimal dosing regimens for other antibiotics and for biofilms grown under different medium conditions or *in vivo*.

These are among the first experimental results, that we are aware of, which demonstrate that providing the optimal break from antibiotics can indeed sensitize biofilms to treatment. The observed U-shaped response curve around the break from antibiotic agrees with theoretical modeling studies on biofilm removal by intermittent application (Cogan et al., 2013, 2016; Zhao et al., 2016; Acar and Cogan, 2019). The optimal length of break likely depends on the time to resuscitation of dormant cells (Carvalho et al., 2018; Butini et al., 2019), which will be a function of both unique effects of the antibiotic used (pharmacodynamics) and the nutrient availability across the biofilm. This concept is similar to that of the post-antibiotic effect (PAE), but is subtly different, as it is the time to resuscitation not to regrowth. We have correlated this with the PK-PD variable of Time below MIC, Time < MIC, not to be confused with the PK-PD parameters of beta-lactams required for growth inhibition or bactericidal activity, which are usually Time > MIC. The planktonic MIC may not be the threshold concentration at which persisters resuscitate in the biofilm, but is used for simplicity as a concentration at which to quantify the antibiotic “break.” We found that under rich medium conditions, a relatively short break from oxacillin was optimal (3–4 h below the planktonic MIC in the broth treating the biofilms). If the break was extended, a population resistant to oxacillin began to expand. This resistant subpopulation may have been able to expand in sub-MIC antibiotic concentrations, and then survive repeated applications of antibiotic by virtue of slow-growth rate or being present in pockets of the biofilm experiencing low concentrations of oxacillin (Lee et al., 2018). Encouragingly, resistant cells did not expand in the optimally sensitizing regimen of oxacillin (Figure 5), suggesting a therapeutic window for biofilm efficacy with periodic break dosing.

Biofilms, and more generally persisters, are notoriously difficult to treat. Treatment strategies shown to activate dormant cells through providing alternative metabolites (Borriello et al., 2006; Meylan et al., 2018; Li et al., 2019), or targeting signaling pathways (Marques et al., 2014; Koo et al., 2017), are promising, as are approaches that use antibiotics that kill dormant cells (Defraine et al., 2018), such as ADEP4, with an ATP independent mechanism to kill dormant *S. aureus* (Conlon et al., 2013), or colistin to kill dormant *P. aeruginosa* (Pamp et al., 2008). Our result of achieving efficacy against biofilms with a single antibiotic by providing periodic breaks is encouraging in its simplicity. More sobering was the narrow window of effective break, and the variability in sensitization, that we observed against *S. aureus* biofilms with oxacillin. This narrow effective window may also explain why intermittent dosing is not necessarily more effective than continuous infusion in animal models of endocarditis (Robaux et al., 2001; Jacqueline et al., 2002). With individual variation in antibiotic PK, a correctly timed periodic break would be challenging to achieve clinically with oral or parental oxacillin treatment. However, potential applications may exist for wound treatment with topical antibiotics or for antimicrobial lock therapy for catheters, where tight control of antibiotic levels is maintained. Increasing the frequency of applied breaks may also improve efficacy, and deserves future exploration. Our experiments were conducted under rich medium conditions with very mature and dense biofilms, and further research is needed to study the effective break period and frequency under alternative conditions. It is intriguing to consider the possibility of removing the antibiotic, supplying a short interval of nutrients to activate the biofilm, and then reapplying antibiotic, achieving greater control over the time to resuscitation of persisters (Acar and Cogan, 2019). Several strategies to increase the range of the break window also suggest themselves, such as sequential treatment with different antibiotics (Butini et al., 2019), or searching for antibiotic pairs that exhibit cellular hysteresis (Roemhild et al., 2018) in regard to persister resuscitation and sensitivity, or exploiting collateral sensitivity to maximize clearance of resistant cells with the second antibiotic (Baym et al., 2016). We expect that the use of compounds such as teixobactin, for which there is no detectable resistance (Ling et al., 2015), will allow for more relaxed regimens with a broader window between antibiotic applications.

Using a clinically approved antibiotic, we were able to achieve efficacy against mature *S. aureus* biofilms by providing short and repeated breaks where the antibiotic was below a critical threshold. Targeting the dormant portion of a biofilm can be achieved by correct timing of dosing, relying on resuscitation during drops in the antibiotic. Biofilms are indeed difficult to treat; their dormancy is their shield, but knowing this allows us to exploit and undermine it.

## Data Availability Statement

The raw data supporting the conclusions of this article will be made available by the authors, without undue reservation.

## Author Contributions

KM and KL designed the study and wrote the manuscript. KM performed the experiments and analyzed results. HT, JS, and MG assisted with experiments and revised the manuscript. All authors contributed to the article and approved the submitted version.

### Conflict of Interest

The authors declare that the research was conducted in the absence of any commercial or financial relationships that could be construed as a potential conflict of interest.
