# Supplementary material for: Pulse Dosing of Antibiotic Enhances Killing of a Staphylococcus aureus Biofilm
Source: Front Microbiol. 2020 Nov 9;11:596227. doi: 10.3389/fmicb.2020.596227 (PMC7680849; doi:10.3389/fmicb.2020.596227)
Supplement: Supplementary file 1 [file Data_Sheet_1.pdf]

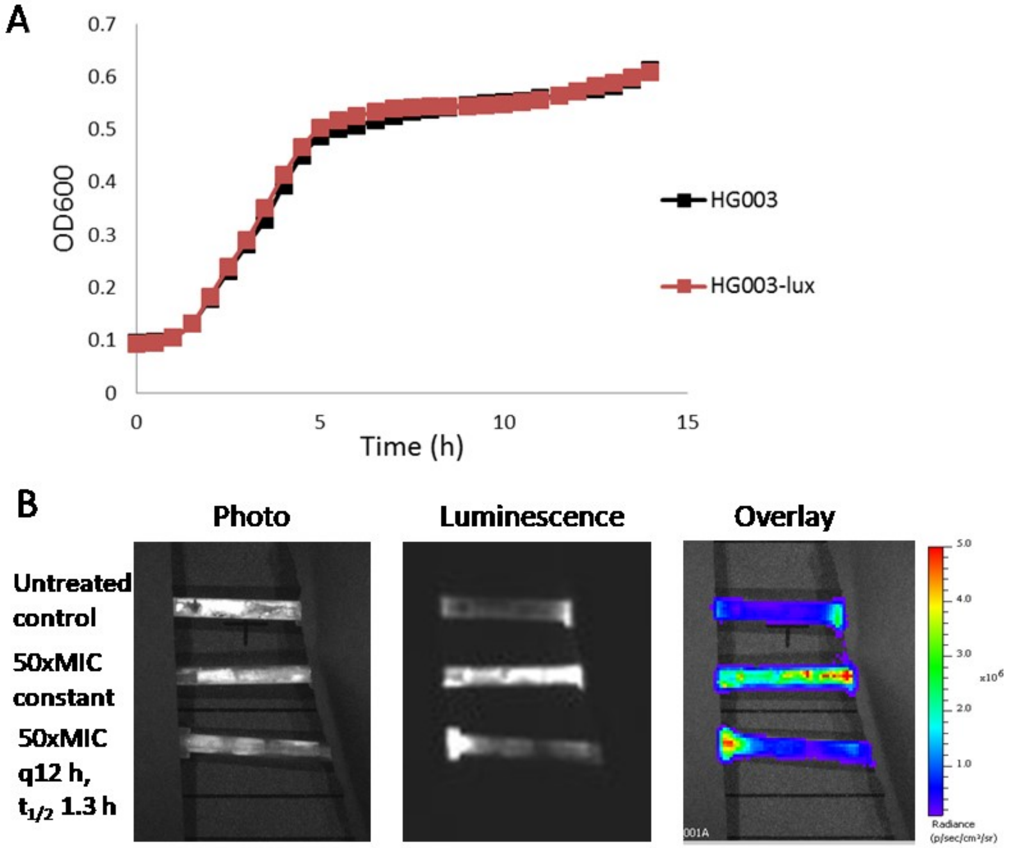

Figure S1. Growth and luminescence of HG003-lux. (A) The HG003-lux strain carrying a chromosomal lux cassette had the same growth as parent strain HG003, determined by triplicate growth curves of each strain grown overnight in a 96-well plate in a plate reader, 37°C with shaking, with OD600 readings every 30 min. **(B)** Segments of the glass tubes carrying the biofilm laden catheters were disconnected from the flow tubing on day 4 of treatment (oxacillin experiment), and imaged for luminescence in an IVIS Lumina LT, Living Image v4.3.1.0.15880. The untreated control has dense biofilms but it has relatively low luminescence suggesting cells are in a slow growth state. Treated biofilms display some luminescence; however, this varied widely across and between experiments. Black paper is laid across the plastic tubing connections to remove noise.

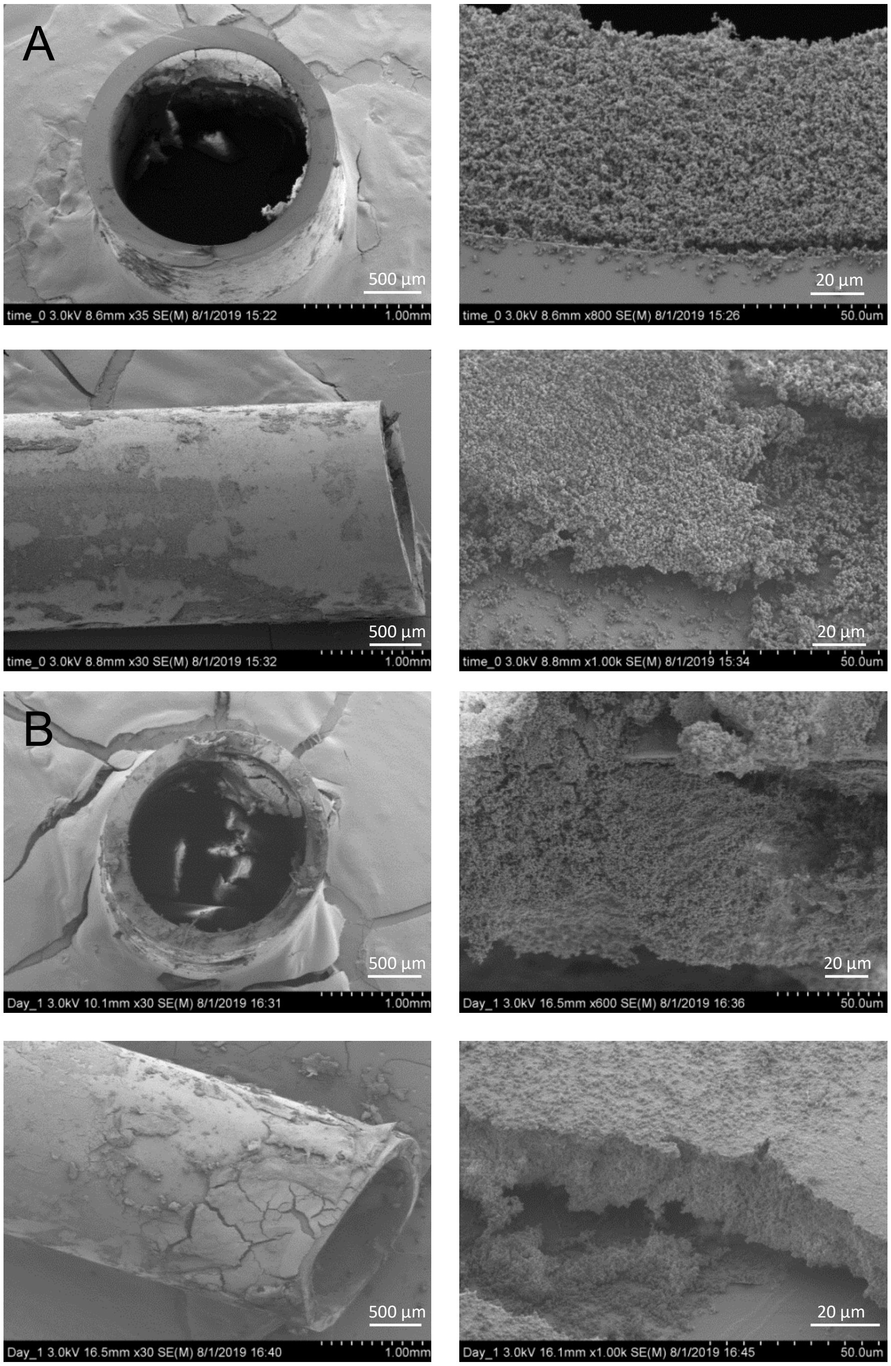

Figure S2. SEM images of *S. aureus* biofilms. Interior and exterior of catheters from the *in vitro* flow system on Day 0 **(A)**, and Day 1 **(B)**. Left and right are different magnification images of the same catheter. SEM preparation removed much of the biofilm on the exterior of catheters, but remnants remained illustrating the thick mass of cells that had coated the catheters.

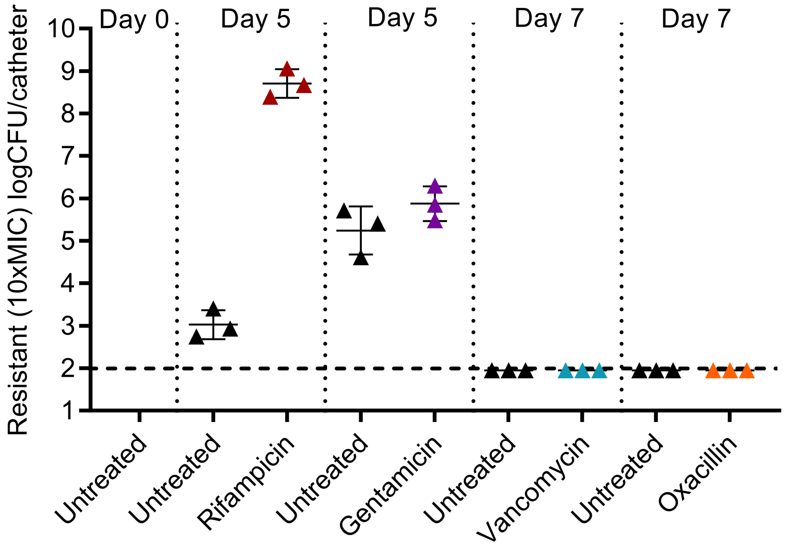

Figure S3. Resistant CFU from biofilms on catheters. Disrupted biofilm suspensions were plated on agar plates containing 10xMIC of relevant antibiotic (rifampicin, gentamicin, vancomycin, or oxacillin) to enumerate resistant CFU, from matched untreated controls or biofilms exposed to 100xMIC of the antibiotic constantly for the specified number of days. Untreated and treated biofilms display substantial growth on gentamicin plates likely due to the subpopulation of small colony variants known to arise in *S. aureus* biofilms, and typically resistant to gentamicin due to reduced proton motive force – these colonies, from both untreated and treated biofilms, did not appear until 48 – 72 h after plating.

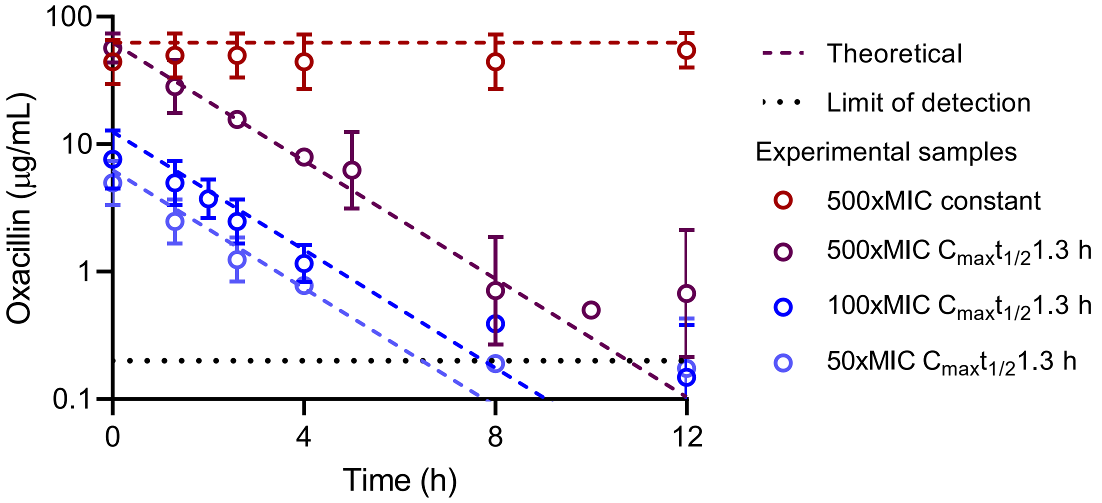

Figure S4. Pharmacokinetic check of oxacillin regimens. Samples were removed from central bottle feeding biofilms and oxacillin concentration was measured using a bioassay against a standard curve of known concentrations. Dashed lines represent the theoretical pharmacokinetics of the modeled regimens, symbols represent means of samples from repeat experiments, $1 \leq n \leq 7$, lines are SD. Dotted line is limit of detection.
